# Medication use among patients with Crohn’s disease or ulcerative colitis before and after the initiation of advanced therapy

**DOI:** 10.1186/s12876-022-02584-4

**Published:** 2022-11-19

**Authors:** Theresa Hunter, April N. Naegeli, Chi Nguyen, Mingyang Shan, Joseph L. Smith, Hiangkiat Tan, Klaus Gottlieb, Keith Isenberg

**Affiliations:** 1 Eli Lilly and Company, 893 S. Delaware Street, Indianapolis, IN 46225 USA; 2 HealthCore, Inc., Wilmington, USA; 3 Anthem, Inc., Indianapolis, USA

**Keywords:** Abdominal pain, Advanced therapies, Crohn’s disease, Ulcerative colitis

## Abstract

**Background:**

Although various treatments help reduce abdominal pain, real-world pain medication utilization among patients with Crohn’s disease (CD) or ulcerative colitis (UC) receiving advanced therapies is poorly understood. The aim is to understand the utilization of pain medication 12 months before and after the initiation of advanced therapies among patients with newly diagnosed CD or UC.

**Methods:**

This retrospective, observational cohort study used administrative medical and pharmacy claims data of patients with CD or UC from HealthCore Integrated Research Database (HIRD^®^). The data from patients with use of pain medication over 12 months follow-up (after the initiation date of advanced therapies) were collected and analyzed. Differences in the use of pain medication 12 months before and after the initiation of advanced therapies were assessed using McNemar's and Wilcoxon signed-rank test.

**Results:**

Prior to initiating advanced therapies, 23.1% of patients with CD (N = 540) received nonsteroidal anti-inflammatory drugs (NSAIDs), 78.1% glucocorticoids, 49.4% opioids, and 29.3% neuromodulators; similarly, 20.9% of patients with UC (N = 373) received NSAIDs, 91.4% glucocorticoids, 40.8% opioids, and 29.5% neuromodulators. After receiving advanced therapies for 12 months, patients reported a reduction in the use of steroids (78.1% vs. 58.9%, *P* < 0.001 in CD; 91.4% vs. 74.3%, *P* < 0.001 in UC), opioids (49.4% vs. 41.5%, *P* = 0.004 in CD; 40.8% vs. 36.5%, *P* = 0.194 in UC), and NSAIDs (23.1% vs. 15.0%, *P* < 0.001 in CD; 20.9% vs. 15.8%, *P* = 0.035 in UC), while the use of neuromodulators significantly increased (29.3% vs. 33.7%, *P* = 0.007 in CD; 29.5% vs. 35.7%; *P* = 0.006 in UC).

**Conclusions:**

The use of pain medications such as NSAIDs, glucocorticoids, opioids, and neuromodulators was common among patients with CD or UC. These results highlight that patients with CD or UC continued to receive pain medications even after initiating advanced therapies.

## Background

Inflammatory bowel disease (IBD) is a chronic, progressive inflammatory disorder of the gastrointestinal tract, and includes Crohn’s disease (CD) and ulcerative colitis (UC) as its 2 major forms [1]. The United States (US) has reported the highest age-standardized prevalence rate, accounting for nearly one-fourth of the global IBD cases [2]. The estimated prevalence of IBD ranges from 252 to 439 cases per 100,000 population in US [3], creating sustained demand on health care delivery systems and communities in treating IBD [4].

Pain due to inflammation during the early phase of IBD, affects 50–70% of patients [5] and contributes to the poor quality of life (QoL) [6]. Chronic abdominal pain is one of the primary presenting symptoms in patients seeking medical care during initial diagnosis or exacerbation of IBD [7, 8]. Although the therapeutic strategy for IBD aims at reducing chronic abdominal pain, a significant number of patients (20–50%) continue experiencing it even during periods of remission [9, 10]. In addition, extra-intestinal manifestations can also cause pain in patients with IBD [11]. Primary inflammatory manifestations in patients with IBD include inflammation of the skin, eyes, and joints. If secondary effects of disease are also considered, nearly 100% of patients with IBD have an abnormality outside of the gastrointestinal tract lumen [12]. The complex associations among pain, anxiety, depression, and QoL observed in multiple IBD cohort studies have established the critical need for effective individualized management of abdominal pain [13].

Treating pain in patients with IBD can be challenging, as commonly used therapies such as nonsteroidal anti-inflammatory drugs (NSAIDs), opioids, and corticosteroids have been associated with an increased risk of disease exacerbation and other adverse outcomes [14, 15]. However, these medications and advanced therapies [e.g., tumor necrosis factor inhibitors (TNFi), non-TNFi, and Janus kinase inhibitors (JAKi)]) provide symptomatic relief and can result in healing of the inflamed intestine in CD or UC conditions [16, 17]. Use of advanced therapies such as TNFi and JAKi have improved the health-related QoL in patients with IBD by decreasing symptomatology associated with hospital admissions and reducing side effects experienced from the use of other conventional therapies [18–20]. Although advanced therapies have been shown to achieve clinical response in patients with CD or UC, their role in reducing the use of other pain medications to alleviate pain symptoms in IBD remains poorly understood.

Though various treatments show promising results in reducing abdominal pain [7], real-world pain medication utilization among patients receiving advanced therapies is not adequately understood [21]. The goal of this study is to better understand the utilization of pain medications 12 months before and after the initiation of advanced therapies among the newly diagnosed patients with CD or UC in commercially insured or Medicare Advantage US populations.

## Methods

### Study design and data source

This retrospective, observational cohort study used administrative medical and pharmacy claims data of patients with CD or UC from the HealthCore Integrated Research Database (HIRD). The HIRD represents a broad, clinically, and geographically diverse spectrum of longitudinal claims data from US patients who are insured through Anthem’s commercial or Medicare Advantage health plans in the Northeastern, Southern, Midwestern, and Western regions. As of Q1 2019, the HIRD contained data of over 51 million individuals dating back to January 1, 2006. The HIRD is updated monthly with fully adjudicated paid claims.

The data were used in full compliance with the relevant provisions of the Health Insurance Portability and Accountability Act of 1996 (HIPAA). The study was conducted under the research provisions of Privacy Rule 45 Code of Federal Regulations (CFR) 164.514(E). This study was conducted in accordance with legal and regulatory requirements, scientific purpose, value and rigor, and follow Good Practices for Outcomes Research issued by the International Society for Pharmacoeconomics and Outcomes Research (ISPOR).

### Patient population

Newly diagnosed adult patients with CD or UC from January 1, 2014 till July 31, 2017 using International Classification of Diseases (ICD), ninth revision, Clinical Modification (CM) (ICD-9-CM: 555.xx for CD; 556.xx for UC) and tenth revision (ICD-10-CM: K50.x for CD; K51.x for UC) codes were included. Other inclusion criteria were ≥ 2 claims for advanced therapies of interest following CD or UC diagnosis between the first diagnosis of CD or UC and the end of the study period, having ≥ 1 year of continuous health plan enrollment prior to the initiation date of advanced therapies (index date), and ≥ 1 year of continuous health plan enrollment after index date.

In patients with CD, the following US Food and Drug Administration (FDA)-approved advanced therapies were included: natalizumab, certolizumab pegol, adalimumab, infliximab, vedolizumab, and ustekinumab. Similarly, in patients with UC, the following FDA-approved advanced therapies were included: adalimumab, infliximab, golimumab, vedolizumab, tofacitinib, and ustekinumab.

Patients with juvenile idiopathic arthritis or those with other/multiple autoimmune conditions such as ankylosing spondylitis, psoriatic arthritis, rheumatoid arthritis, and systemic lupus erythematosus were excluded. Also, patients with both CD and UC were excluded from the study.

### Study outcomes

Data on demographic and clinical characteristics, and the use of pain medication over 12 months follow-up (after the index date), were collected and analyzed.

Any chronic, unclassified pain condition (general pain, fibromyalgia, pelvic pain, abdominal pain, muscle pain, or myalgia) was identified during baseline period and classified according to Romanelli et al. [22]. The number and percentage of patients with ≥ 1 medical claim(s) for the above-mentioned conditions were reported.

Pain medications included NSAIDs, glucocorticoid steroids, non-narcotic analgesics, opioids (chronic opioid use ≥ 180 days), and neuromodulators (antidepressants, anticonvulsants, muscle relaxants). The frequency or percentage of patients with ≥ 1 fills and number of fills of each pain medication listed were reported during the baseline period and first year after the index date.

Adherence to advanced therapies was measured by the proportion of days covered (PDC) as defined in literature [23]. The PDC was calculated as the number of days with drug on-hand or number of days exposed to drug divided by the number of days in the specified time interval. Adherence was defined as PDC ≥ 80% [23]. PDC was assessed during 12 months after the initiation of advanced therapies. The change in pain medication fills in both CD and UC cohorts was reported 12 months before and 12 months after the initiation of advanced therapies.

### Statistical analysis

Continuous variables (demographics, clinical characteristics 12 months before and 12 months after initiating advanced therapies) were presented as mean and standard deviation values. Categorical variables such as pain medication use before and after initiating advanced therapies were presented as relative frequencies and percentages. McNemar’s and Wilcoxon signed-rank tests were used to compare changes in pain medication use before and after the initiation of advanced therapies. Generalized estimating equation models were used to examine the total number of pain medication fills in CD and UC cohorts over time. These models controlled for patient demographics, clinical characteristics, and accounted for within-subject variations over time. As the outcome was over-dispersed, negative binomial regression with log link functions and unstructured correlation was selected based on model fit. Determination of model baseline covariates (including demographics, comorbid conditions, and adherence to advanced therapies) were based on clinical importance and finalized after review of the univariate analysis.

## Results

### Demographic and clinical characteristics

**Fig. 1:**
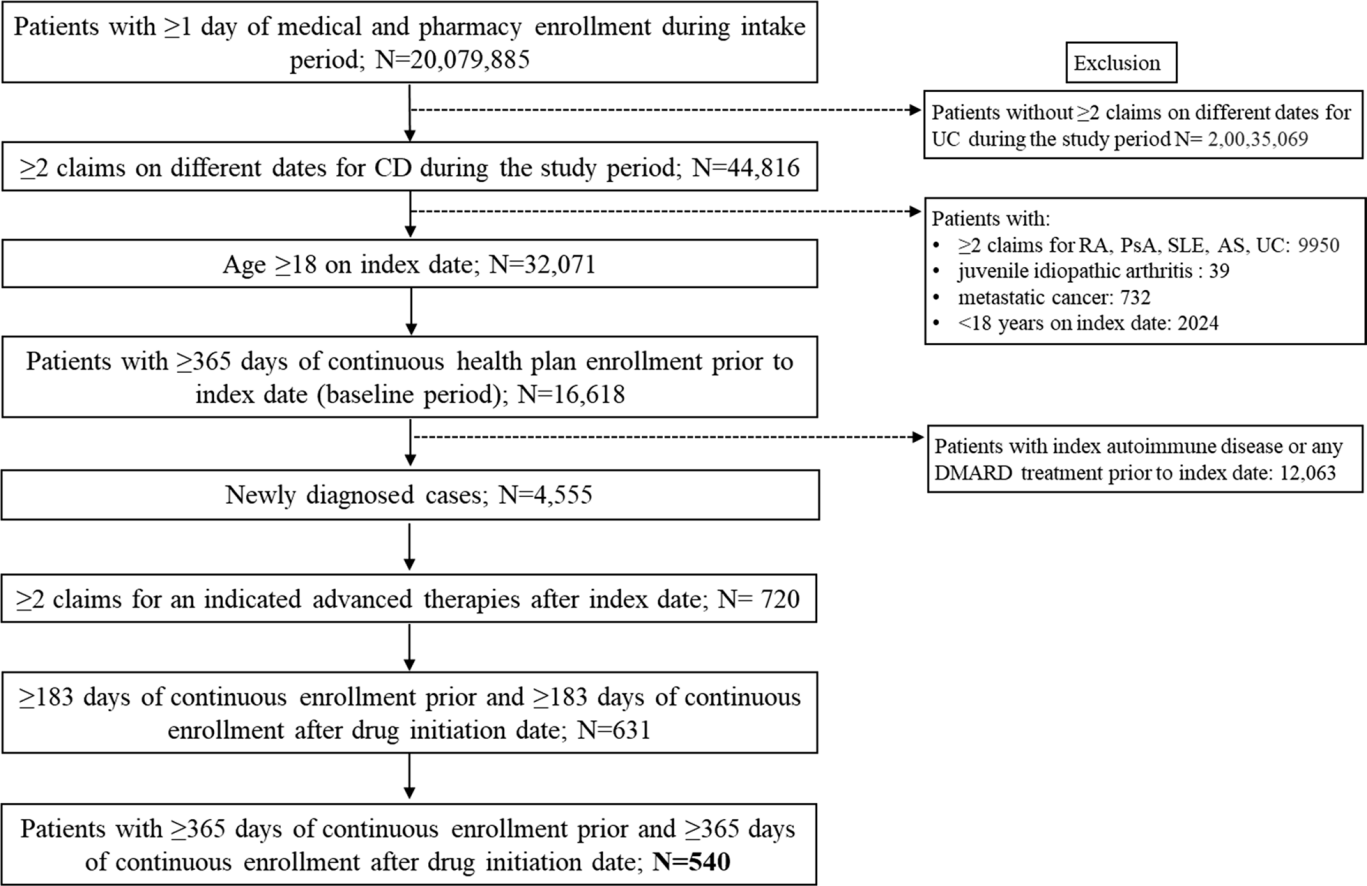
Study disposition and inclusion of patients in CD cohort. *AS* ankylosing spondylitis, *CD* Crohn’s disease, *DMARD* disease-modifying antirheumatic drug, *PsA* psoriatic arthritis, *RA* rheumatoid arthritis, *SLE* systemic lupus erythematosus, *UC* ulcerative colitis

**Fig. 2:**
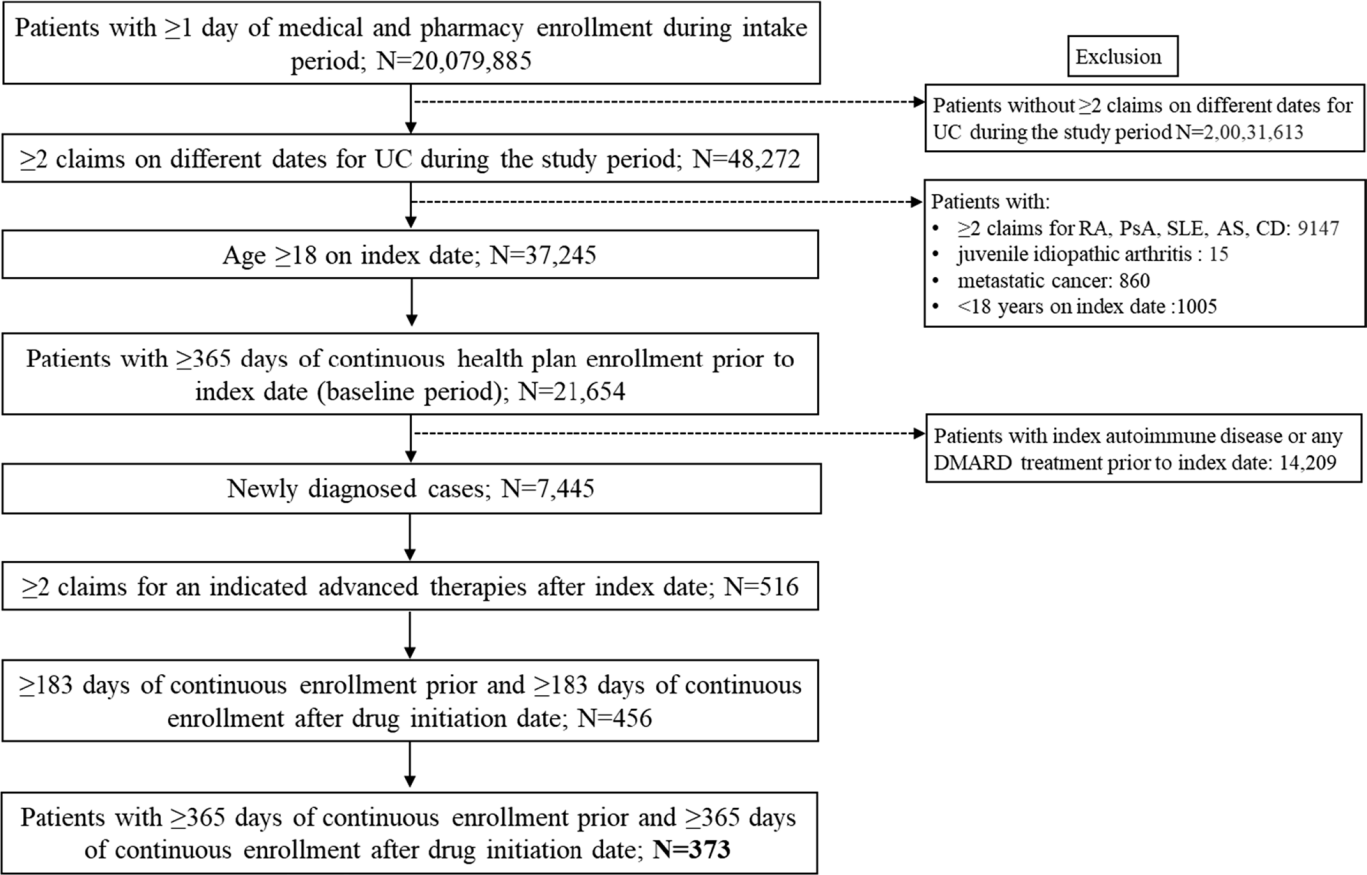
Study disposition and inclusion of patients in the UC cohort. *AS* ankylosing spondylitis, *CD* Crohn’s disease, *DMARD* disease-modifying antirheumatic drug, *PsA* psoriatic arthritis, *RA* rheumatoid arthritis, *SLE* systemic lupus erythematosus, *UC* ulcerative colitis

The analyses included 540 and 373 patients with CD and UC, respectively (Figs. 1, 2).

Baseline demographics and clinical characteristics of the patients included are presented in Table 1.

**Table 1:** Baseline demographic and clinical characteristics

	CD	UC
	N = 540	N = 373
Age on index date (years)		
Mean (SD)	36.8 (14.6)	39.9 (15.2)
Age categories, n (%)		
18–34	275 (50.9)	161 (43.2)
35–44	96 (17.8)	82 (22.0)
45–54	86 (15.9)	58 (15.5)
55–64	69 (12.8)	49 (13.1)
≥ 65	14 (2.6)	23 (6.2)
Sex, n (%)		
Female	270 (50.0)	164 (44.0)
Residence region, n (%)		
Northeast	88 (16.3)	62 (16.6)
Midwest	160 (29.6)	104 (27.9)
South	192 (35.6)	122 (32.7)
West	94 (17.4)	84 (22.5)
Plan type, n (%)		
HMO	141 (26.1)	79 (21.2)
PPO	299 (55.4)	232 (62.2)
CDHP	100 (18.5)	62 (16.6)
Other/unknown	0 (0)	0 (0)
Index diagnosis, n (%)		
2014	142 (26.3)	103 (27.6)
2015	175 (32.4)	114 (30.6)
2016	141 (26.1)	109 (29.2)
2017	82 (15.2)	47 (12.6)
Time from index diagnosis to the initiation date of advanced therapies (months)		
Mean (SD)	7.1 (9.1)	10.9 (11.1)
Provider specialty on index date, n (%)		
Gastroenterologist	166 (30.7)	128 (34.3)
Primary care physician	35 (6.5)	28 (7.5)
Others	334 (61.9)	217 (58.2)
QCI		
Mean (SD)	0.4 (0.8)	0.5 (1.0)
Comorbid conditions, n (%)		
Peripheral vascular disease	16 (3.0)	11 (2.9)
Chronic pulmonary disease		61 (11.3)
Mild liver disease		49 (9.1)
Diabetes without chronic complication	24 (4.4)	32 (8.6)
Anemia	183 (33.9)	134 (35.9)
Dyslipidemia	71 (13.1)	79 (21.2)
Fibromyalgia	14 (2.6)	*
Hypertension	105 (19.4)	88 (23.6)
Mental illness	201 (37.2)	118 (31.6)
Osteoarthritis	35 (6.5)	28 (7.5)
Infections	291 (53.9)	207 (55.5)
Nicotine dependence/tobacco use disorder	121 (22.4)	72 (19.3)

*CD* Crohn’s disease, *CDHP* Consumer Directed Health Plan, *QCI* Quan-Charlson Comorbidity Index, *SD* standard deviation, *UC* ulcerative colitis, *HMO* Health Maintenance Organization, *PPO* Preferred Provider Organization, *ref* reference

*Denotes a cell value ≤ 10 that was blinded for privacy reasons

The mean ± SD age of patients with CD and UC were 36.8 ± 14.6 years and 39.9 ± 15.2 years, respectively. Most incident cases with CD (50.9%) and UC (43.2%) belonged to the age category of 18–34 years. A total of 50% of patients in the CD cohort were females and 44% were females in the UC cohort. Majority of the patients with CD (35.6%) or UC (32.7%) were from the Southern region of the US. The mean time from diagnosis to the initiation of advanced therapies was 7.1 months for the CD cohort and 10.9 months for the UC cohort.

### Pain medication use

**Fig. 3:**
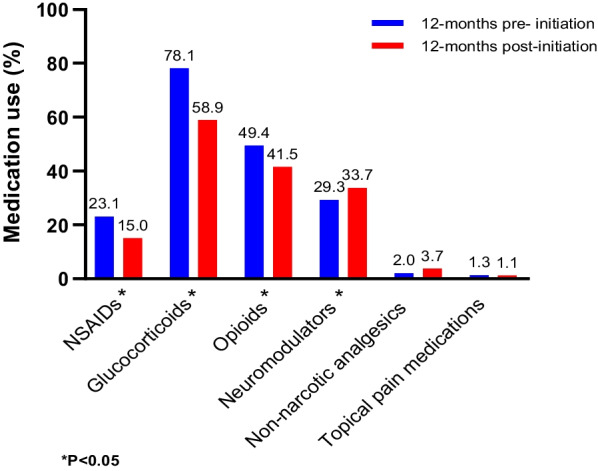
Use of pain medication for CD: before and after the initiation of advanced therapies. *CD* Crohn’s disease, *NSAIDs* nonsteroidal anti-inflammatory drugs. *P-value calculated using McNemar's test for categorical variables and paired t-test or Wilcoxon signed-rank test for continuous variables

**Fig. 4:**
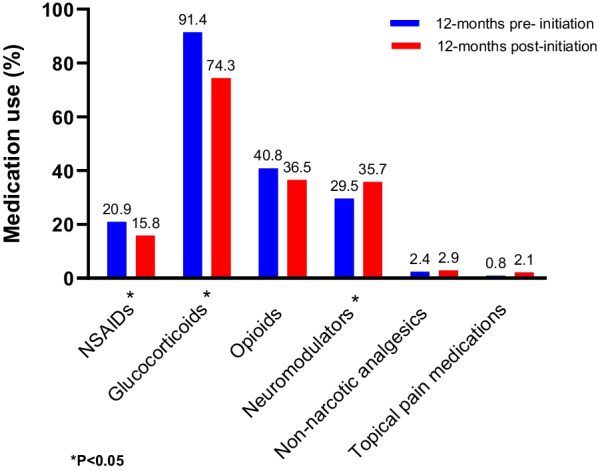
Use of pain medication for UC: before and after the initiation of advanced therapies. *NSAIDs* nonsteroidal anti-inflammatory drugs, *UC* ulcerative colitis. *P-value calculated using McNemar's test for categorical variables and paired t-test or Wilcoxon signed-rank test for continuous variables

Before the initiation of advanced therapies, 23.1% of patients with CD were prescribed NSAIDs, 78.1% glucocorticoids, 49.4% opioids, and 29.3% neuromodulators; similarly, 20.9% of patients with UC received NSAIDs, 91.4% glucocorticoids, 40.8% opioids, and 29.5% neuromodulators (Figs. 3, 4).

However, 12 months after the initiation of advanced therapies, there was a significant reduction in the use of glucocorticoids (78.1% vs. 58.9%, *P* < 0.001 in CD; 91.4% vs. 74.3%, *P* < 0.001 in UC), opioids (49.4% vs. 41.5%, *P* = 0.004 in CD; 40.8% vs. 36.5%, *P* = 0.194 in UC), and prescription-based NSAIDs (23.1% vs. 15.0%, *P* < 0.001 in CD; 20.9% vs. 15.8%, *P* = 0.035 in UC), while the use of neuromodulators significantly increased (29.3% vs. 33.7%, *P* = 0.007 in CD; 29.5% vs. 35.7%, *P* = 0.006 in UC) (Figs. 3, 4).

### Number of pain medication fills

In the CD cohort, the generalized estimating equation models showed that there was no significant change in pain medication fills/administrations among patients who adhered to advanced therapies (exponentiated estimate [EE]: 0.89; *P* = 0.0638). On the other hand, non-adherent patients had 17% more pain medication fills/administrations (EE: 1.17; *P* = 0.0375; Table 2) when compared to the initiation of advanced therapies before 12 months.

**Table 2:** CD cohort: number of pain medication fills/administrations before and after advanced therapy initiation (GEE model)

	Exponentiated estimate	95% confidence interval	*P* value
Advanced therapy PDC (ref = 0, before initiation)			
PDC 0–0.8 (nonadherence)	1.17	1.01, 1.35	0.0375
PDC ≥ 0.8 (adherence)	0.89	0.79, 1.01	0.0638
Baseline arthritis/joint pain (ref = no)	0.97	0.78, 1.19	0.7502
Baseline back/cervical pain (ref = no)	1.24	1.01, 1.53	0.0432
Baseline neuropathies/neuralgias (ref = no)	1.28	1.02, 1.62	0.0358
Baseline migraines/headaches (ref = no)	1.23	0.93, 1.62	0.1507
Baseline unclassified pain (ref = no)	1.05	0.85, 1.29	0.6571
Age (ref = 18–34)			
35–44	1.32	1.09, 1.59	0.0043
45–54	1.23	0.96, 1.56	0.0996
55–64	1.00	0.79, 1.27	0.9873
≥ 65	0.95	0.52, 1.73	0.8610
Female (ref = male)	1.27	1.09, 1.47	0.0019
Residence region (ref = Northeast)			
Midwest	1.17	0.93, 1.46	0.1832
South	1.05	0.85, 1.30	0.6693
West	1.05	0.81, 1.37	0.7087
Other/unknown	0.94	0.34, 2.61	0.9042
Plan type (ref = HMO)			
PPO	0.97	0.82, 1.16	0.7573
CDHP	0.90	0.71, 1.13	0.3490
Medicare Advantage or Supplement coverage (ref = no)	1.38	0.70, 2.73	0.3588
At initiation year (ref = 2014)			
2015	0.94	0.74, 1.20	0.6249
2016	0.89	0.69, 1.15	0.3677
2017	0.83	0.64, 1.08	0.1619
2018	0.56	0.37, 0.85	0.0062
Duration from index diagnosis to advanced therapy initiation (months)	1.01	1.00, 1.02	0.1467
QCI (ref = 0)			
1	1.37	1.10, 1.71	0.0045
2	1.11	0.88, 1.39	0.3718
3+	0.92	0.52, 1.64	0.7848
Other baseline conditions			
Anemia (ref = no)	1.13	0.97, 1.31	0.1172
Dyslipidemia (ref = no)	1.31	1.05, 1.64	0.0178
Fibromyalgia (ref = no)	1.69	1.22, 2.32	0.0014
Hypertension (ref = no)	1.30	1.07, 1.59	0.0093
Mental illness/substance use disorders (ref = no)	2.03	1.72, 2.39	< 0.0001
Osteoporosis (ref = no)	1.20	0.47, 3.07	0.7060
Infections (ref = no)	1.16	1.00, 1.35	0.0547
Nicotine dependence (ref = no)	0.87	0.72, 1.05	0.1419

Model adjusted for baseline pain conditions, age, gender, geographic region, plan type, Medicare Advantage coverage, duration of index diagnosis to advanced therapies initiation, QCI, and other baseline conditions

*CD* Crohn’s disease, *CDHP* Consumer Driven Health Products, *GEE* generalized estimating equations, *HMO* Health Maintenance Organization, *PDC* proportion of days covered, *PPO* Provider Preferred Organization, *QCI* Quan-Charlson Comorbidity Index, *ref* reference

The number of pain medication fills were significantly associated with presence of baseline conditions such as back/cervical pain (EE: 1.24, 95% confidence interval [CI] 1.01–1.53, *P* = 0.0432), neuropathies/neuralgias (EE: 1.28, 95% CI 1.02–1.62, *P* = 0.0358), middle age group (35 to 44 years; EE: 1.32, 95% CI 1.09–1.59, *P* = 0.0043), and female patients (EE: 1.27, 95% CI 1.09–1.47, *P* = 0.0019).

In the UC cohort, it was observed that after controlling the baseline factors, there was no significant change in pain medication fills/administrations among adherent (EE: 0.95; *P* = 0.3668) and non-adherent patients (EE: 1.15; *P* = 0.0769; Table 3) after initiating advanced therapies.

**Table 3:** Advanced UC cohort: number of pain medication fills/administrations before and after advanced therapy initiation (GEE model)

	Exponentiated estimate	95% confidence interval	*P* value
Advanced therapy PDC (ref = 0, before initiation)			
PDC 0–0.8 (nonadherence)	1.15	0.98, 1.35	0.0769
PDC ≥ 0.8 (adherence)	0.95	0.85, 1.06	0.3668
Baseline arthritis/joint pain (ref = no)	1.22	0.99, 1.50	0.0563
Baseline back/cervical pain (ref = no)	1.02	0.82, 1.27	0.8367
Baseline neuropathies/neuralgias (ref = no)	1.01	0.79, 1.28	0.9612
Baseline migraines/headaches (ref = no)	1.34	1.00, 1.80	0.0504
Baseline unclassified pain (ref = no)	1.23	0.93,1.61	0.1426
Age (ref = 18–34)			
35–44	1.31	1.04, 1.65	0.0223
45–54	1.31	1.02, 1.67	0.0312
55–64	1.27	0.98, 1.65	0.0661
≥ 65	1.37	0.89, 2.11	0.1491
Female (ref = male)	1.09	0.93, 1.29	0.2851
Residence Region (ref = Northeast)			
Midwest	0.97	0.76, 1.25	0.8274
South	1.13	0.90, 1.42	0.3104
West	1.23	0.95, 1.59	0.1216
Other/unknown	1.85	1.36, 2.51	< 0.0001
Plan type (ref = HMO)			
PPO	0.94	0.78, 1.13	0.4925
CDHP	0.88	0.68, 1.14	0.3353
Medicare Advantage or Supplement coverage (ref = no)	0.76	0.46, 1.26	0.2840
At initiation year (ref = 2014)			
2015	1.08	0.72, 1.61	0.7209
2016	1.05	0.71, 1.57	0.7989
2017	1.03	0.68, 1.55	0.8915
2018	0.90	0.55, 1.45	0.6537
Duration from index diagnosis to advanced therapy initiation (months)	1.00	0.99, 1.01	0.7598
QCI (ref = 0)			
1	0.90	0.68, 1.21	0.4911
2	1.10	0.84, 1.44	0.4843
3+	1.22	0.89, 1.68	0.2247
Other baseline conditions			
Anemia (ref = no)	1.05	0.89, 1.24	0.5703
Dyslipidemia (ref = no)	1.11	0.89, 1.39	0.3499
Fibromyalgia (ref = no)	0.75	0.27, 2.08	0.5804
Hypertension (ref = no)	1.40	1.13, 1.73	0.0022
Mental illness/substance use disorder (ref = no)	1.64	1.38, 1.94	< 0.0001
Osteoporosis (ref = no)	1.15	0.78, 1.70	0.4806
Infections (ref = no)	1.18	0.99, 1.40	0.0649
Nicotine dependence (ref = no)	1.05	0.86, 1.27	0.6412

Model adjusted for baseline pain conditions, age, gender, geographic region, plan type, Medicare advantage coverage, duration of index diagnosis to advanced therapies initiation, QCI, and other baseline conditions

*CDHP* Consumer Driven Health Products, *GEE* generalized estimating equations, *HMO* Health Maintenance Organization, *PPO* Provider Preferred Organization, *PDC* proportion of days covered, *QCI* Quan-Charlson Comorbidity Index, *UC* ulcerative colitis

The age of patients (35 to 44 years; EE: 1.31, 95% CI 1.04–1.65, *P* = 0.0223; 45 to 54 years; EE: 1.31, 95% CI 1.02–1.67, *P* = 0.0312 as compared with a younger group of 18 to 34 years) and presence of other baseline conditions such as hypertension (EE: 1.40, 95% CI 1.13–1.73, *P* = 0.0022) and mental illness/substance use disorders (EE: 1.64, 95% CI 1.38–1.94, *P* < 0.0001) were associated with higher number of pain medication fills.

## Discussion

This study provides real-world information on the treatment patterns for the use of medication to reduce pain in patients with CD or UC during the 12-month period before and after the use of advanced therapies. Our findings suggested that patient prescriptions filled for pain medications decreased across both disease cohorts after 12 months compared with before 12 months of initiation of advanced therapies. Despite the reduction, the usage of pain medication remained relatively high across both disease cohorts. Prior to the initiation of advanced therapies, steroids were among the most common medications used, followed by opioid pain treatment in both CD and UC cohorts.

Corticosteroids were the preferred choice to treat CD; whereas 5-aminosalicylic acids (5-ASA) followed by corticosteroids were the most common initial treatment for UC [24]. While the effectiveness of corticosteroids, NSAIDs, and opioids in relieving pain is documented [25], they are associated with several serious adverse drug reactions such as exacerbating gut symptoms and bowel dysmotility [26], limiting their long-term use. A significant number of patients use opioids or marijuana for pain control despite the psychological and disease-related risks [27].

A recent study using the Truven MarketScan Commercial Claims and Encounters database has shown that an increasingly larger proportion of patients were treated with advanced therapies from 2007 to 2015 (CD: 21.8–43.8%; UC: 5.1–16.2%) relative to more stable use of immunomodulators or 5-ASA [28] and similar to patterns observed in other studies using claims data [29, 30]. Other studies suggest that patients are much more likely to follow non-biologic treatment pathways compared with advanced therapies treatment pathways for CD (81% vs. 19%; *P* < 0.05, t-test) and UC (94% vs. 6%; *P* < 0.05, t-test) [24]. We observed similar trends wherein newly diagnosed patients were more likely to utilize pain medications over advanced therapies.

The current analysis corroborates the patterns observed in previous work where the first advanced therapy was initiated in CD and UC cohorts after the use of corticosteroids and immunomodulators [24]. We observed that the use of steroids and NSAIDs decreased significantly in 12 months after the initiation of advanced therapies, whereas the use of neuromodulators increased in both the cohorts. Although the use of these pain medications decreased after the initiation of advanced therapies, their use was still prevalent among patients. We observed that 41.5% of patients with CD and 36.5% of patients with UC received opioids; 58.9% and 74.3% of patients with CD and UC, respectively, were prescribed corticosteroids after initiating advanced therapies. Since opioids have overarching effects on pain regulation regardless of the source of pain, their chronic use has been reported in 30% of patients with IBD, referred for psychiatric evaluation [31]. On the other hand, the frequency of opioid use ranged from 3 to 13% in all the patients presenting to an IBD clinic [32, 33], much lower than the prevalence reported here. Opioids can complicate IBD, as increasing doses of narcotics will cause or aggravate the pain that is being treated and increase the risk of narcotic bowel syndrome characterized by chronic abdominal pain [6]. The increased use of other neuromodulators could be an indication of efforts to decrease the use of opioids. Only 45% of newly diagnosed patients with UC required corticosteroids [34], compared with the current data wherein more than half of the patients with both CD (58.9%) and UC (74.3%) continued their use. The prevalence of steroids use even after the initiation of advanced therapies indicates that the patients were not in corticosteroid-free remission. Although patients continued with other therapies for pain, the pain medication fills significantly decreased in adherent patients 12 months after the initiation of advanced therapies in CD.

Of the approved advanced therapies for CD or UC, TNFi and α4β7 integrin receptor antagonists were commonly used [35, 36]. In our study, most of the patients with CD (95.0%) or UC (89.3%) used TNFi during the 12 months of advanced therapy use in our study (data not shown), confirming prior findings [35, 36]. Irrespective of the symptoms/disease condition, it has also been reported that these drugs can be highly effective whether administered as monotherapy or in combination with pain medications early in the disease course. In both CD and UC, poor disease control with prior therapies is one reason for the initiation of advanced therapies [37]. Studies cited here and supported by this analysis show that the management of IBD has evolved in the last decade to increasingly rely on escalating pharmacotherapies, particularly advanced therapies.

To our knowledge, this is the first study reporting claims-based real-world data on pain medication use by patients with CD or UC on pain medication use before and after the initiation of advanced therapies. The current data highlight the prevalence of pain medication use in newly diagnosed patients with CD or UC.

This study has some limitations. It was not possible at all instances to know the circumstances for which patients were using pain medications (e.g., flaring disease, abdominal pain in the absence of active condition). Although we assessed pain medication use among patients with IBD, we cannot ascertain if the pain medication was prescribed for IBD specifically. Thus, it was not possible to stratify patients according to disease activity or severity. Although we assessed pain medication use among patients with IBD, over-the-counter pain medication use was not assessed. We cannot ascertain if the pain medication was prescribed for IBD specifically. The study population was limited to US patients who were insured (Anthem’s commercial or Medicare Advantage), which could impact the applicability of the results to other populations (traditional Medicare, Medicaid, uninsured populations, or non-US patients). There may be errors in the diagnosis of disease as coded on claims recorded in the databases. We did not include over-the-counter use of pain medication and had the short follow-up period of 12 months after the initiation of advanced therapies which does not address long-term trends in disease course and pain treatment therapies.

## Conclusions

In summary, the findings reported here suggest that use of pain medications such as NSAIDs, glucocorticoids, opioids, and neuromodulators is common among patients with CD or UC. Additionally, the results highlight that those patients with CD or UC continued to receive pain medications even after initiating advanced therapies. These findings are clinically relevant because they suggest the complexity of IBD pain management. These data expand the literature on pain medication utilization for IBD abdominal pain, especially the role of advanced therapies. Future research is needed to develop better treatment plans for abdominal pain in IBD.

## Data Availability

The HealthCore database is a proprietary health claims database and is not accessible to the public. HealthCore researchers were provided access pursuant to HIPAA. Further information concerning access to the HealthCore database may be provided upon request.

## Abbreviations

5-ASA: 5-Aminosalicylic acids
CD: Crohn’s disease
CFR: Code of Federal Regulations
CI: Confidence interval
CM: Clinical modification
EE: Exponentiated estimate
FDA: Food and Drug Administration
HIPAA: Health Insurance Portability and Accountability Act
HIRD: HealthCore Integrated Research Database
ICD: International Classification of Diseases
IBD: Inflammatory bowel disease
IRB: Institutional Review Board
ISPOR: International Society for Pharmacoeconomics and Outcomes Research
JAKi: Janus kinase inhibitors
NSAID: Nonsteroidal anti-inflammatory drugs
PDC: Proportion of days covered
QoL: Quality of life
TNFi: Tumor necrosis factor inhibitors
UC: Ulcerative colitis
US: United States
